# Elderly or ageless? Physical Activity in the Aged Orthopaedic Patient

**DOI:** 10.3390/jcm9103243

**Published:** 2020-10-10

**Authors:** Fabio Pigozzi, Vincenzo Denaro

**Affiliations:** 1 Department of Movement, Human and Health Sciences, University of Rome “Foro Italico”, 00100 Rome, Italy; 2 Department of Orthopaedic and Trauma Surgery, Campus Bio-Medico University of Rome, 00128 Rome, Italy; denaro@unicampus.it

Progression of osteoarthritis in the elderly is often a synonym of impaired function, discontinuation of physical activity and sport participation [1]. However, there is consistent evidence suggesting that the role of sports activity is of paramount importance in the whole natural history of osteoarthritis, as well as of degenerative disc disease. This contrast should be promptly faced by the orthopaedic surgeon, gerontologist and sports physician, to avoid early impairment or discontinuation of patient’s activity. In fact, apart from preventing the onset of major symptoms and delaying the loss of function as primary and secondary prevention, exercise is extremely important to improving muscular conditioning and strength before surgery and for the post-operative recovery and rehabilitation.

According to the World Health Organization, the concept of physical activity refers to several entities, including light individual exercise, collective training, individual or team sports participation [2]. All of these activities have specific effects on the whole organism and allow the human body to remain healthy not only for musculoskeletal fitness but also for the improvement of cardiovascular, metabolic and psychosocial status. If this is especially true for young people, the elderly become ageless if they are able to stay fit, to stay healthy and to maintain the physical and mental fitness that allow them to face organ deterioration, functional impairment and possible major surgeries. From an economical and occupational perspective, it is worth underlining that, given the increased retirement age of citizens in Europe, preserving a good to excellent functional status is of paramount importance to improving productivity and avoiding early retirement and inability to work [3]. All of these features are enclosed within the framework of successful ageing, according to which the organism deterioration follows an ordered pathway to avoid patient discomfort and disability [4].

Therefore, physical activity represents complementary therapeutics for the management of osteoarthritis and for other musculoskeletal degenerative diseases. A special focus of international research concerns the involvement of older patient in exercise programs as a conservative treatment, but also in the preoperative setting, to improve surgical outcomes [5]. It is a common experience for the orthopaedic surgeon that a fit patient has a faster and better recovery after a major surgical procedure, including joint arthroplasty, and next to clinical experience, the literature evidence is growing, reporting significant improvement of patient-reported outcomes either for pain or for function [6].

The aim of the present Special Issue is to collect the available evidence concerning the role of physical activity as a conservative treatment for large joint osteoarthritis and low back pain, and as prehabilitation and rehabilitation, before and after arthroplasty surgery. A systematic approach has been followed for evidence collection, and a meta-analytic methodology has been advocated in most of the papers, to ensure a comprehensive assessment of available data.
